# Analytical evidence of enamel hypomineralisation on permanent and primary molars amongst past populations

**DOI:** 10.1038/s41598-017-01745-w

**Published:** 2017-05-10

**Authors:** Elsa Garot, Christine Couture-Veschambre, David Manton, Cédric Beauval, Patrick Rouas

**Affiliations:** 1 Univ. de Bordeaux, UFR des Sciences Odontologiques, Bordeaux, France; 2 Univ. de Bordeaux, PACEA, UMR 5199, Pessac, France; 3 Melbourne Dental School, University of Melbourne, Victoria, Australia; 4 Archéosphère, 2 Rue des Noyers, 11500 Quirbajou, France

## Abstract

Molar Incisor Hypomineralisation (MIH) and Hypomineralised Second Primary Molars (HSPM) involve prevalent qualitative structural developmental anomalies of tooth enamel affecting the first permanent molars (and often incisors) and the second primary molars, respectively. These demarcated hypomineralised lesions of enamel manifest as white-cream or yellow-brown opacities, with possible post-eruptive localised loss of enamel. Aetiological hypotheses have involved contemporary life factors (i.e. environmental pollutant exposure or early childhood medications) in contrast to factors not limited to a specific time period (i.e. hypoxia at birth or genetic predisposition). Evidence of MIH in ancient populations would reinforce aetiological factors present for many centuries. By means of microtomographic and X-ray fluorescence analyses the present study provides evidence that (i) two archaeological specimens: “S407” (Sains-en-Gohelle, France, 12^th^–16^th^ centuries) and “B335” (Beauvais, France, 15^th^–18^th^ centuries) were MIH-affected, and (ii) one individual “S323” was affected by HSPM and MIH (Sains-en-Gohelle, France, 7^th^–11^th^ centuries).

## Introduction

Teeth contribute to human bodily functions such as mastication, phonation, appearance and maxillofacial development. Enamel is the most highly mineralised bodily tissue, and thus in an archaeological context is the best-preserved^1^. Teeth have been a focus of interest for physical anthropologists over many generations. Teeth provide much information about humans including cultural environment, locational migration, pathology, morphological variation, age estimation and sex differentiation^2^. Pathologies, such as enamel hypoplasia and dental caries, are informative for understanding the health and nutritional status of individuals and populations^3, 4^. A modified Developmental Defects of Enamel (DDE) index classifies enamel anomalies, with enamel demarcated opacities separated into two subgroups: white-cream and yellow-brown^5^. In 2001, Molar Incisor Hypomineralisation (MIH)^6^ was defined as a qualitative demarcated enamel hypomineralisation defect of tooth enamel affecting at least one first permanent molar (FPM), often affecting permanent incisors^6^. More recently, the term Hypomineralised Second Primary Molar (HSPM) was used to describe similar defects affecting second primary molars^7^. The presence of HSPM increases the risk of MIH, but the absence of HSPM does not exclude the presence of MIH^8–10^. The European Academy of Paediatric Dentistry agreed on MIH diagnosis criteria characterised by at least one of these factors affecting one or more FPMs: demarcated enamel opacity, post-eruptive enamel breakdown, atypical restoration, or atypical extraction due to MIH^11^. Post-eruptive enamel breakdown is defined as a defect that indicates a decrease in the depth of enamel after eruption of the tooth. Loss of initially formed surface enamel after tooth eruption is often associated with a pre-existing demarcated opacity^11^. Today, the worldwide prevalence of MIH is between 2.9 and 44% (average 15%) of children based on current population studies^12^. Given the difficulties involved in treating MIH (hypersensitivity, child anxiety, difficulties with anaesthesia, poor aesthetics, carious lesions with fast progression, failure of restorations), it is essential that the aetiology is determined in order to allow risk assessment and early diagnosis, and if possible, prevention of risk factors^13, 14^. Without early diagnosis, post-eruptive structural damage may occur quickly and eventually lead to FPM extraction. Currently, in the absence of identified cause(s), no risk prevention actions can be implemented. MIH constitutes a public health problem, with consequences that are not only health-related but also economic. MIH may impact on the well-being of young patients in a crucial period of infant development^15, 16^. The proposal for a single term “MIH” and the establishment of specific diagnosis criteria by consensus promoted and encouraged the multiplication and quality of research works, but some issues remain unresolved^11^. Amongst the aetiological hypotheses mentioned in the literature, some recently introduced factors such as pollutants (dioxin derivatives^17^ or Bisphenol^18^) or drugs^19^ (antibiotics^20–22^ or asthma drugs^23^) have been proposed. Other hypotheses of putative factors that have occurred over time, such as childhood illness (in particular fever), prematurity, hypoxia at birth or a genetic predisposition, are also mentioned regularly^24–29^. Most studies are retrospective in nature and there is currently insufficient published evidence to identify specific aetiological factors relevant to MIH^30^, but authors agree a multifactorial aetiology is likely^27^. The relationship between the occurrence of HSPM and MIH suggests common risk factors^9^. The identification of MIH and HSPM amongst ancient populations would highlight that aetiological factors apart from the proposed contemporary factors have relevance. Except for a few anthropological studies^31–33^, demarcated enamel hypomineralisation has only been observed in individuals from the 20^th^ and 21^st^ centuries. Anthropological researchers have diagnosed MIH in historical populations based on clinical diagnosis^31–33^. To date, no author has identified an HSPM case in a past population.

In an archaeological context, McKay and colleagues demonstrated, by means of microcomputed tomography, that brown enamel opacities caused by taphonomic contamination can be confused with hypomineralisation^34^, as chemical elements (*e.g*. Iron or Manganese) contained in the burial ground can stain teeth^35, 36^ (*post mortem*) and the resultant staining can be similar in appearance to discoloration caused by MIH^37, 38^ (*ante mortem*). Before concluding that MIH was present in past populations, it is necessary to discriminate between discolouration caused by pathological (*ante mortem*) or taphonomic (*post mortem*) processes^37^.

Here, two historic osteological series (Sains-en-Gohelle and Beauvais, France) selected owing to a significant number of children aged 6 to 18 years were assessed. Using a diagnostic guide^37^, teeth showing defects indicative of MIH and HSPM, including respectively, 298 and 14 individuals aged from 6 to 18 years were analysed. Three individuals affected by yellow-brown opacities on first permanent molars, incisors or second primary molars were selected for analysis: B335 (Beauvais, France, 15^th^–18^th^ centuries), S323 (Sains-en-Gohelle, France, 7^th^–11^th^ centuries) and S407 (Sains-en-Gohelle, France, 12^th^–16^th^ centuries). After distribution of shared high resolution images securely via the internet, clinical diagnoses of the three individuals were given by nineteen MIH specialists. This study aimed, by means of enamel microanalyses, to confirm or refute these clinical diagnoses of MIH and HSPM and discuss their implications.

## Results

### Collection examination

**Figure 1:**
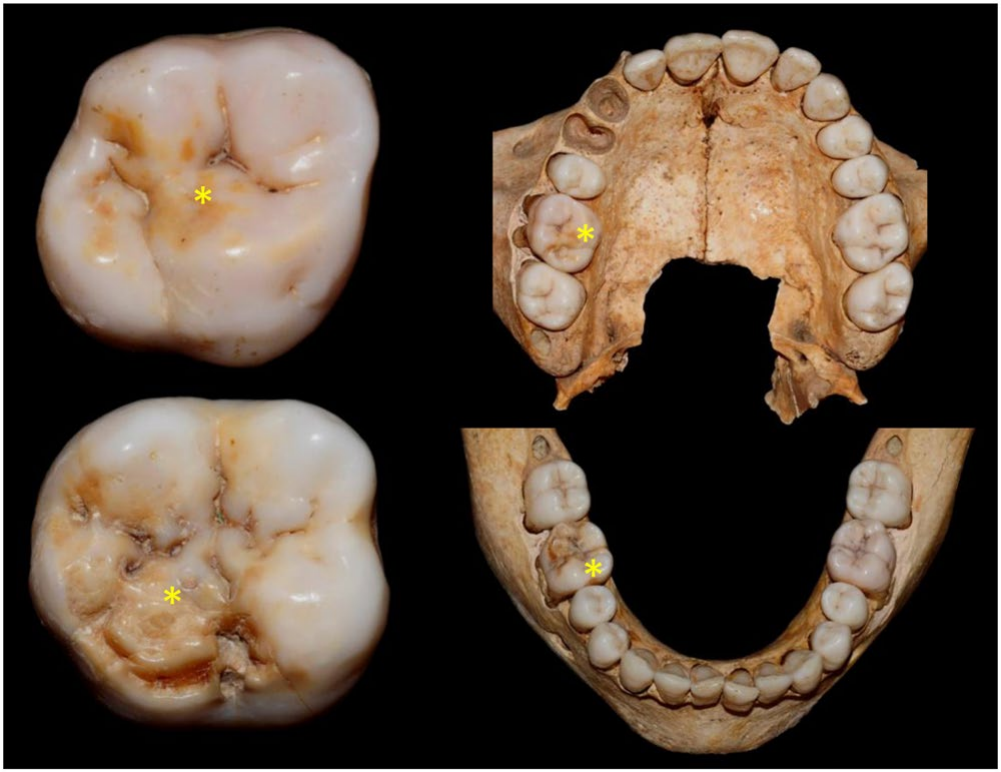
Photographs of materials fro﻿m B335 (Beauvais, France). *teeth affected by discolorations.

**Figure 2:**
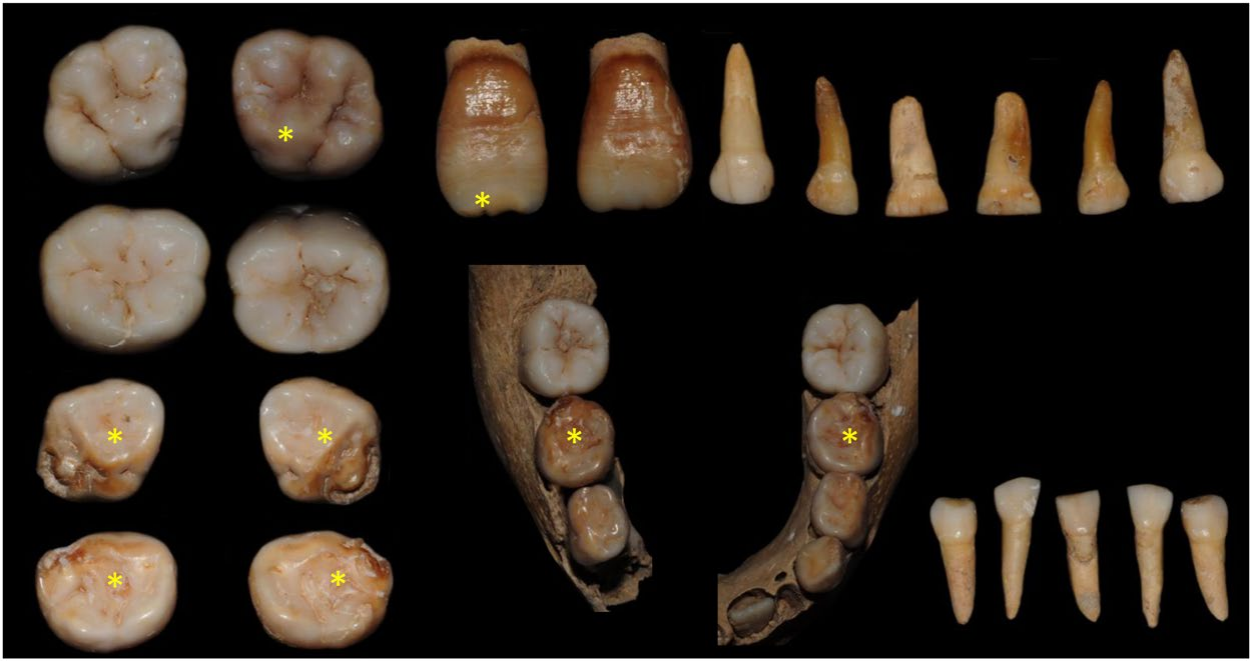
Photographs of materials from S323 (Sains-en-Gohelle, France). *teeth affected by discolorations.

**Figure 3:**
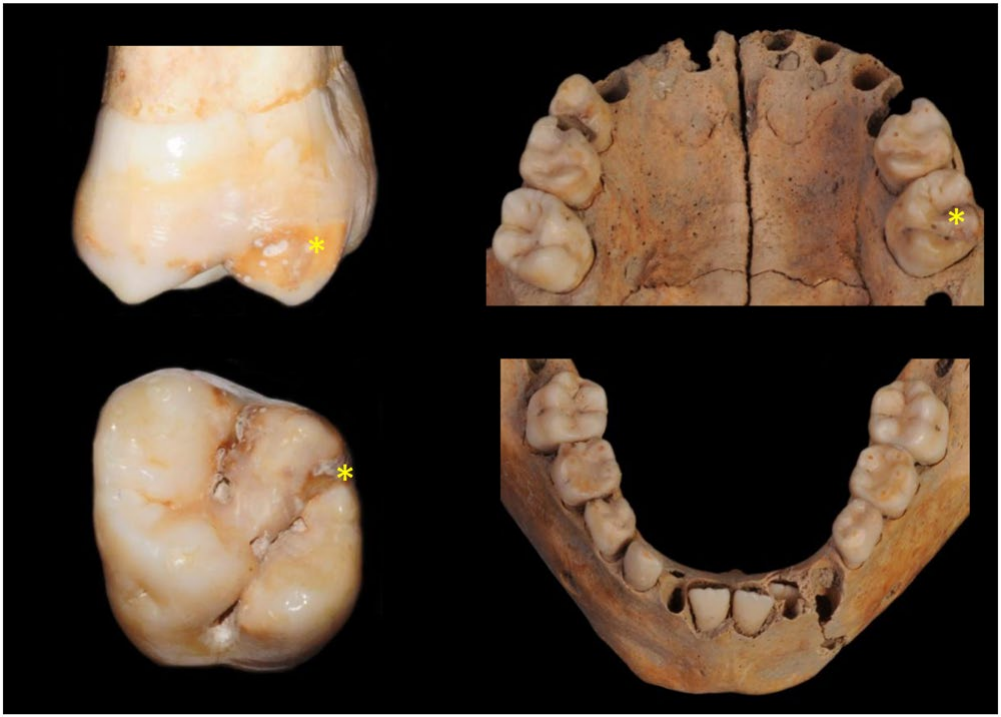
Photographs of materials from S407 (Sains-en-Gohelle). *teeth affected by discolorations.

The anthropological study of the **B335, S323** and **S407** materials highlighted opacities and post-eruptive enamel breakdown appearing similar to MIH and HSPM (Figs 1–3). The maxilla and mandible of **B335** from the Beauvais series are stored at the bone library of Pessac (PACEA Lab, UMR5199, University of Bordeaux, France)^39^. This individual was a teenager, aged 14 years 5 months (95% CI between 10 years and 5 months to 18 years 5 months). All permanent teeth were present at death and a brown demarcated opacity was observed on tooth 16 (Fig. 1). The remaining maxillary dentition showed neither discoloration, carious lesions, nor wear (sound). There was *post mortem* loss of teeth 13 and 14. Regarding the mandibular teeth, post-eruptive breakdown (PEB) of enamel with brown discoloration was present on tooth 46. The other mandibular teeth were sound.

The individuals **S323 and S407** are from the Sains-en-Gohelle series dated to the medieval period (confirmed by mean of radiocarbon analysis of individuals)^40^. The material from **S323** included the first permanent incisor germs, the first permanent molars and the primary molars. Age was estimated at 3 years and 9 months to 6 years^40^ (Fig. 2). Some teeth showed similar patterns of MIH and HSPM, such as tooth 16 with a brown discoloration in the occlusal third of the coronal surface, the four second primary molars exhibited brown opacities with PEB in the occlusal third and tooth 11 had creamy discoloration of the incisal edge (Fig. 2). A linear hypoplastic lesion of enamel is observed in the cervical third of tooth 11. The second primary mandibular molars show mild occlusal wear. The other first permanent molars (26, 36 and 46) were sound. Specimen **S407** (Fig. 3) remains included the maxilla, the mandible and teeth including the first permanent molars and first and second primary molars. Age was estimated at 3 years and 4 months to 6 years^40^. A yellow demarcated opacity was present on the occlusal third of the buccal surface of tooth 26. No other discolorations were observed but some carious lesions on primary molars were present (teeth 54, 55, 64, 65, 84 and 85) and slight occlusal wear was observed on the primary molars.

### MIH experts’ diagnoses

Photographic images of the three specimens (Figs 1–3) were examined by 19 MIH specialists (see Methods and SI). Concerning B335, seven MIH experts did not confirm the MIH diagnosis and one of them refuted this diagnosis. Only eight experts confirmed the MIH diagnosis for S323. Scores were better correlated for S407 because 14/19 specialists gave an MIH diagnosis. The Fleiss’ kappa test performed for each specimen showed a poor agreement between responses (κ < 0) concerning MIH diagnosis (SI).

### Microcomputed tomographic (µCT) analyses

**Figure 4:**
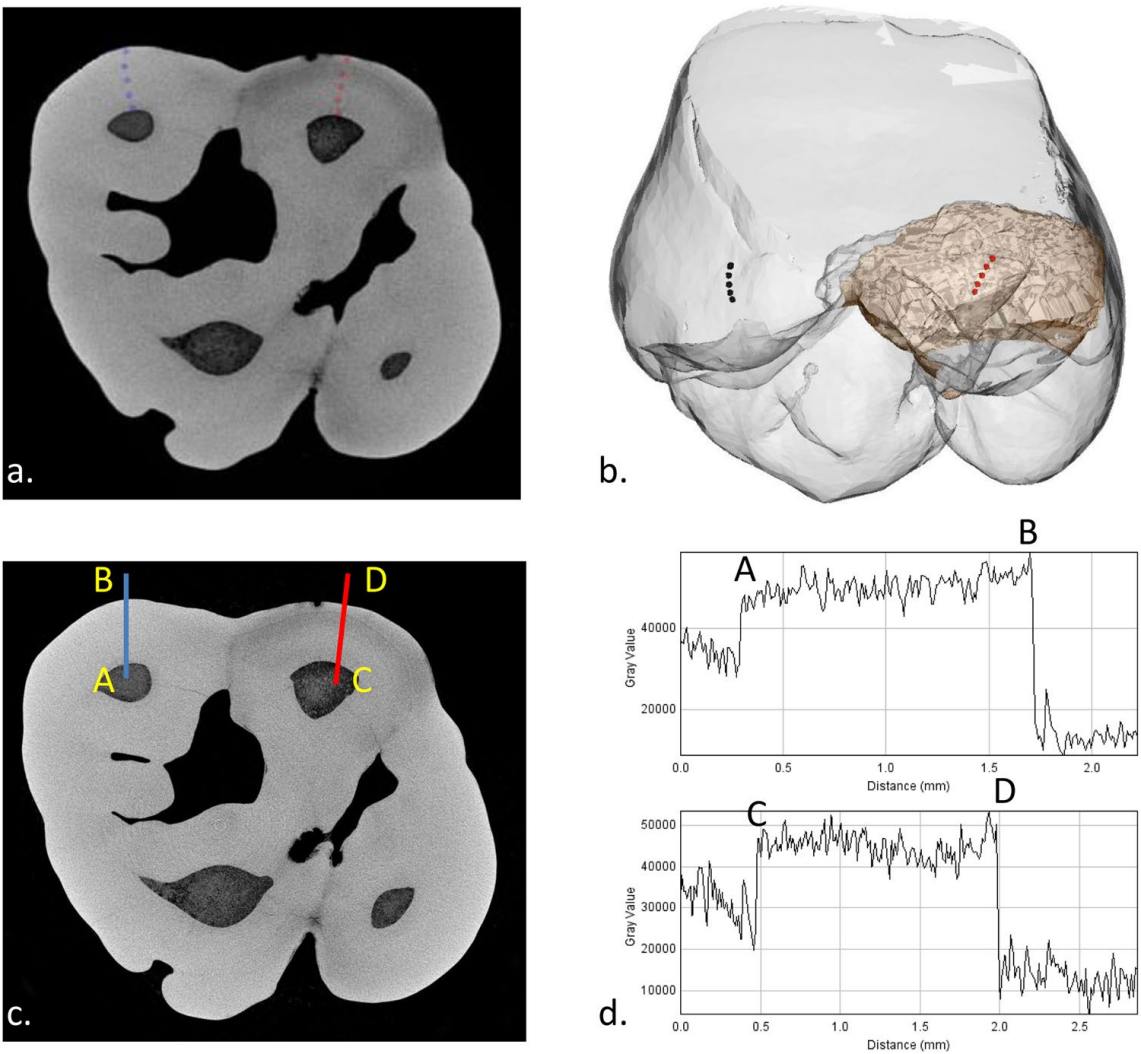
Images of microcomputed tomography analyses in 3D and 2D: (**a,b**) location of 5 analysis cubes from DEJ to surface on normal (blue cubes) a﻿nd discoloured (red cubes) areas. (**c,d**) location of profile lines from EDJ to surface through normal (AB) and discoloured (CD) areas.

Mineral densities of discoloured and normal enamel areas at the same coronal height for each tooth were measured from µCT data in 3D (Fig. 4a) and 2D (Fig. 4b; see Methods; SI); summarised in Table 1. Except for S323(11), all teeth with discolorations had significantly lower mineral densities in stained enamel (P < 0.05). Amongst the control samples including normal teeth [B335(36), S323(16) and S323(21)], no significant differences in mineral density were detected (P > 0.1; Table 1).

**Table 1:** Wilcoxon test for paired samples performed from 3D data between normal and discoloured enamel among discoloured teeth (a), and between normal areas of enamel among control sample (b). Analyse 1 was repeated a second time (analyse 2).

Ind (n°tooth)		Analyse 1 (GL)					Analyse 2 (GL)					P value
		mdd1	mdd2	mdd3	mdd4	mdd5	mdd1	mdd2	mdd3	mdd4	mdd5	
a.	B335(46)	4082	4453	1453	740	−357	7038	4531	4323	2843	2396	0.007*
	B335(16)	4071	3948	3337	2600	2300	351	1458	1363	894	2167	0.005*
	S323(26)	60	2329	2639	2554	1013	1652	6173	7293	5612	3312	0.005*
	S323(11)	1059	829	−243	−1380	−1628	−6879	−561	−528	−914	−784	0.1
	S323(55)	2099	5799	3928	518	1575	571	5704	4161	1711	2591	0.005*
	S323(65)	1357	1652	4712	5793	3665	1540	262	4219	5173	1879	0.005*
	S323(75)	417	3800	3628	2468	1523	2329	3461	6010	2553	−1826	0.013*
	S323(85)	1859	4557	6108	4982	2594	1723	476	1570	1799	1371	0.005*
	S407(26)	5381	7887	4428	3378	2321	4395	6955	3385	3090	3131	0.005*
b.	B335(36)	−731	−198	447	776	−384	172	−163	−813	−583	−492	0.2
	S323(16)	−1029	−723	−548	−298	134	1264	1323	1184	1319	123	0.3
	S323(21)	−63	218	594	655	−232	−720	−707	−176	−538	366	0.6

Ind, individual; GL, Grey Level; mdd, mineral density difference between the two enamel areas. *Statistically significant (P < 0.05).

**Figure 5:**
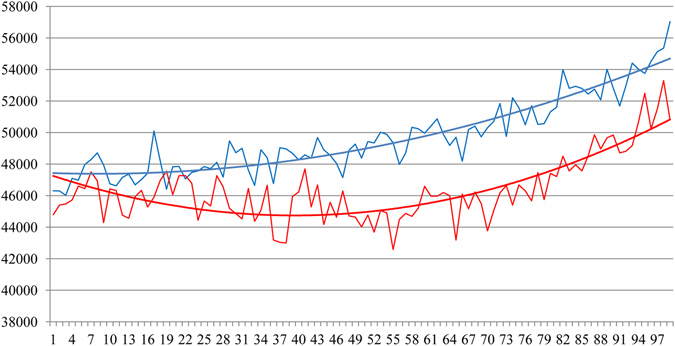
Mineral densities of normal (blue line) and hypomineralised (red line) enamel from 99 equidistant measurements taken on line from DEJ to surface including the discoloured sample (n = 8 with the exception of S323(11)) and the control sample (n = 3).

Mineral concentration line profiles from Dento-Enamel Junction (DEJ) to surface through apparently normal enamel (line AB) and through a discoloured region (line CD) in the discoloured teeth group (n = 8 with the exception of S323(11)) and the control group (n = 3) are illustrated in Fig. 5 (and SI). A decreasing mineral concentration of up to 7% in the middle enamel region along line CD compared with the apparently normal enamel along line AB is apparent in Fig. 5 (and SI). Also, in hypomineralised enamel lesions there is a reverse mineral density gradient with highest density at the DEJ and lowest towards the surface, the opposite of normal enamel.

### X-ray fluorescence analyses

Elemental chemical analyses (see Methods) provided information on taphonomic contamination by one or several chemical elements (Mn, Fe, Cu and Pb). The discolorations on teeth 65 and 75 from S323 were characterised by an increase in Mn (respectively, P = 0.01 and 0.04; Table 2). An increase in Fe was observed in the discoloured area of tooth 46 from B335 (P = 0.01). Other teeth did not show evidence of a taphonomic contamination by these elements (P > 0.05).

**Table 2:** Relative concentrations of Mn, Fe, Cu and Pb and Wilcoxon test for paired sample performed between discoloured (DC) and normal (NO) enamel among discoloured teeth.

Sample	Chemical elements	Mn		Fe		Cu		Pb		P value
		DC	NO	DC	NO	DC	NO	DC	NO	
B335(46)	Mean	**0.072**	**0.030**	**0.189**	**0.026**	**0.011**	**0.011**	**0.104**	**0.048**	0.01*
	SD	0.088	0.029	0.232	0.019	0.004	0.011	0.087	0.036	
B335(16)	Mean	**0.048**	**0.090**	**0.051**	**0.025**	**0.024**	**0.016**	**0.100**	**0.101**	0.18
	SD	0.039	0.132	0.064	0.022	0.015	0.012	0.086	0.132	
S323(26)	Mean	**0.034**	**0.024**	**0.018**	**0.013**	**0.007**	**0.005**	**0.044**	**0.009**	0.21
	SD	0.018	0.016	0.020	0.013	0.008	0.001	0.053	0.006	
S323(11)	Mean	**0.055**	**0.051**	**0.045**	**0.052**	**0.006**	**0.007**	**0.012**	**0.015**	0.40
	SD	0.039	0.037	0.055	0.065	0.002	0.000	0.007	0.007	
S323(55)	Mean	**0.771**	**0.071**	**0.093**	**0.012**	**0.008**	**0.011**	**0.155**	**0.032**	0.05
	SD	0.824	0.035	0.117	0.009	0.001	0.009	0.205	0.035	
S323(65)	Mean	**0.567**	**0.047**	**0.234**	**0.056**	**0.020**	**0.004**	**0.169**	**0.038**	0.01*
	SD	0.408	0.036	0.317	0.046	0.015	0.001	0.179	0.049	
S323(75)	Mean	**0.384**	**0.062**	**0.762**	**0.038**	**0.007**	**0.005**	**0.041**	**0.013**	0.04*
	SD	0.331	0.054	1.020	0.031	0.001	0.000	0.024	0.013	
S323(85)	Mean	**0.134**	**0.023**	**0.147**	**0.052**	**0.005**	**0.004**	**0.029**	**0.038**	0.07
	SD	0.091	0.001	0.156	0.051	0.000	0.001	0.010	0.049	
S407(26)^a^	Mean	**0.063**	**0.023**	**0.092**	**0.049**	**0.004**	**0.004**	**0.005**	**0.008**	0.12
	SD	0.031	0.001	0.019	0.002	0.001	0.001	0.0002	0.001	

SD, Standard deviation; ^a^data from an unpublished study. *Statistically significant (P < 0.05).

## Discussion and Conclusion

Mineral density analyses suggest that the three studied specimens showed evidence of demarcated enamel hypomineralisation on first permanent molars. Moreover, for one specimen (S323), demarcated enamel hypomineralisation on second primary molars is present. A mean mineral density decrease of 7% was calculated at the midway between the DEJ and the surface. Studies highlighted decreased mineral density in hypomineralised areas from 5 to 28% in contemporary hypomineralised teeth^41–47^, with the lower difference possibly due to mineral gain from soil-based minerals incorporated in porous enamel. However, mineral densities of normal enamel discoloured by addition of soils elements were comparable to the mineral densities of unaffected teeth^34^. Similarly to previous studies on contemporary MIH teeth^43–47^, the present results show a constant increase of mineral density in normal enamel from DEJ to surface contrary to a reverse gradient in hypomineralised enamel^43, 45–47^. This MIH feature excludes a possible taphonomic process such as enamel dissolution in acidic burial soil inducing demineralisation starting at the enamel surface^38^. In addition, in the present study, X-ray fluorescence highlighted *post mortem* incorporation of burial components (*i.e*. Fe or Mn) in some samples (B335 and S323), putatively explained by increased porosity of hypomineralised enamel^41, 44^.

All of these signs clearly point to the most ancient case of MIH with HSPM in an osteological collection dated to the 7^th^–11^th^ centuries. This is the first study investigating the structure of discoloured enamel structure to confirm MIH and HSPM diagnoses. No reports of HSPM in past populations have been published; however, authors have reported “probable” MIH in historical populations. A collection of a London cemetery (Broadgate), including individuals suffering from rickets, dated from the 17^th^–18^th^ centuries was previously studied^31, 48^. Scrutiny of the results highlighted a number of points to be clarified. In 2007, these authors reported prevalence for hypoplasia of 93.2% amongst 41 sub-adults, confirmed in some cases by scanning electron microscope. The hypoplastic enamel included 63.6% of molars with severe or moderate lesions^31^. One year later, in another publication, the authors reattributed all the molar teeth with hypoplastic enamel to MIH, and claimed MIH prevalence of 93.2% with 63.6% of molars “showing moderate or severe lesions” amongst the samples^48^. The MIH prevalence of the studied population (93.2%) appears to be very high in comparison to current data (2.9–44%)^12^. Enamel microanalysis for mineral density and elemental profile as well as µCT analysis to allow the determination of enamel thickness would allow the enamel hypoplasia to be distinguished from “probable” MIH. Another case of ‘probable’ MIH was reported in teeth from a skull dating from the mid-15^th^ century^32^. As a result of unusual localization of brown discolorations (*i.e*. first and second premolars, second and third molars) and molar wear, further investigations are required to confirm whether this is MIH or taphonomic stain. A substantial study involving the clinical examination of three archaeological series (dated from 12^th^ to 20^th^ centuries) reported prevalence of 3.1% for MIH and 30.3% for linear enamel hypoplasia^33^. As mentioned by the authors, ‘mix-ups’ between wear (attrition, erosion and abrasion), nutrition, *pre* and *post mortem* discolouration, MIH and other enamel defects in archaeological series are more than probable^33^; the reason why confirmation of diagnosis by microanalyses is important. The present results regarding the uncertainty of visual diagnosis by MIH experts showed that no clinical diagnosis of MIH in past populations is reliable. In fact, authors should confirm their results by means of non-destructive microanalyses^37^.

The discovery of the oldest specimen (dated to the 7^th^–11^th^ centuries) reported to be affected by MIH and HSPM confirmed by microanalyses raises questions about the importance of modern aetiological hypotheses such as medications or environmental pollutants. Several authors have raised the possibility of a link between taking antibiotics during the first years of life and the occurrence of MIH^21, 22, 49–51^. However, the subsequent discovery of the first antibiotic (penicillin) in 1928 and its commercialisation in France in 1945^52^ negates a possible link with MIH and HSPM amongst our sample. This is consistent with Ghanim’s study showing no link between MIH and antibiotics^20^. Noting that in these retrospective studies, it is not possible to identify the real causative factors such as childhood illness (putatively high fever) or the associated medication or both. The hypothesis of environmental dioxins has also been raised in the literature^53, 54^. In fact, authors proposed that dioxins delivered via maternal breastfeeding could be a causative factor of MIH^53^. However, these compounds have only been present in the environment since 1874^55^. The majority of international studies have not established links between MIH and dioxins^17, 49, 50, 56, 57^. More recently, another modern aetiological factor hypothesis has arisen, the bisphenols^18^; used as base compounds in the manufacturing of polycarbonates and epoxy resins. Bisphenol A (BPA) was used worldwide in plastic containers (such as baby bottles and food containers) and since prohibition in France (2015), BPAs were replaced by bisphenol analogues (BPS and BPF)^58^. The commercialisation of BPA took place in 1950^59^, which is not compatible with a possible link with the cases of MIH and HSPM living in the medieval period. None of these modern aetiological hypotheses can explain the enamel defects on the three investigated individuals. If the possible aetiological factors which may have occurred during the 7^th^–18^th^ centuries are studied, childhood illness and *peripartum* events seem to be most plausible. Diseases such as otitis media^50^, pneumonia^49, 50^, infections of the urinary tract^60^, or chicken pox^21^ have been positively associated with MIH. A systematic review investigating 25 studies on MIH aetiological factors determined a probable link with childhood disease^27^. It has also been suggested that the causative factor of MIH could have been a lack of oxygen occurring during deliveries^29, 61–63^. Hypoxia can be associated with medical problems at birth, such as prematurity, caesarean sections^29^, respiratory difficulties and prolonged duration of labour^61^. Other studies have also implicated prematurity in MIH occurrence^23, 50, 61, 64^. More recently a genetic predisposition in conjunction with one or several others factors has also been proposed^24, 26^. Prospective studies should provide more complete temporal results but they are long and complex to carry out. It would take at least six years and the eruption of the FPM in order to collect the initial results, and the initial sample must be large enough to meet the challenges of frequent medical monitoring over a long period (from the third trimester of pregnancy until the age of six to seven years). The first publication using this methodology provides limited information confirming aetiological hypotheses^54^. Pending the results of prospective studies, the increase of MIH prevalence study numbers on ancient populations, including well documented archaeological series, will improve understanding of the MIH pathophysiology.

Thus, the discovery of the oldest specimens with MIH and the first archaeological specimen with HSPM in an immature individual from archaeological series dated from 7^th^–18^th^ centuries indicates similar aetiological factors existing now and in the medieval period such as hypoxia during deliveries, prematurity or childhood diseases, without excluding genetic predisposition. But it should be borne in mind that the aetiology of MIH may be multifactorial. The repeat of observations of a significant prevalence of MIH in older populations would confirm our observations, downplaying somewhat the role of uniquely modern aetiological factors (derivatives of dioxin, bisphenols, antibiotics). The present study highlights the potential contribution of bio-archaeological studies on a current public health problem.

## Methods

### Collection assessment

Individuals with estimated ages from 6 to 18 years old were studied from two archaeological series (Beauvais and Sains-en-Gohelle, France). Only young individuals were chosen owing to the potential for early breakdown of enamel caused by dental caries or wear in archaeological materials in older individuals. The diagnosis of MIH requires erupted FPM in individuals 6 years and older^65^. Some precautionary principles were applied; individuals with taphonomic discoloration of the mandible or maxilla and individuals showing discoloration on all teeth were excluded. Three individuals presenting the most complete dental remains and showing enamel defects indicative of MIH and/or HSPM were selected for analyses: B335, S323 and S407. The first individual (B335) was retrieved from the cemetery of the convent “Les soeurs Grises”, located in Beauvais (France, 15^th^–18^th^, SI). The convent was occupied by the religious community between 1480 and the late 18^th^ century^39, 66^. The age at death of B335 was estimated using the Liversidge and Marsden method^67^. The other specimens (S323 and S407) are from the Sains-en-Gohelle cemetery (France, 7^th^–17^th^ centuries, SI)^40^. The age at death of these two specimens was estimated by means of the Moorrees method^68^.

### Clinical diagnosis

The individuals’ teeth were examined by means of a hand magnifying lens with artificial lighting. Standardised photographs of the three specimens showing brown discolorations similar to MIH and HSPM were taken with a Nikon^®^ (Tokyo, Japan) SLR camera D90, a Metz^®^ (NJ, USA) macro ring flash and a Tamron^®^ (Saitama, Japan) lens SP AF 90 mm f/2.8. Each maxilla, mandible and teeth were photographed on a black matt paper: occlusal view for bones and five views (labial, mesial, lingual, distal and occlusal) for each tooth.

A questionnaire asking for each specimen (B335, S323 and S407) “is it typical of MIH lesions?” and “How did you make your decision?” including photographs of the three specimens was created. It was sent on 23 February 2016 to 76 MIH specialists by means of ‘Google forms’ (Google^®^, CA, USA). The reliability of MIH and HSPM diagnosis by means of photographs were previously validated^69^. Participants selected were authors who had published on MIH in either first or last position in an international journal with an impact factor or in the journal of the EAPD (European Academy of Paediatric Dentistry). A search was performed on 15 February 2016 on Medline/Pubmed with the words “enamel AND hypomineralisation” and 76 authors were identified. From this group, 19 participants answered. The Fleiss’ kappa test was performed between the three possible responses (yes, no and unsure) for each specimen. In a recent published diagnostic guide it was advised that enamel microanalyses should be undertaken to avoid taphonomic bias and MIH misdiagnosis^37^. Therefore, the authors recommended using X-ray fluorescence analyses aiming to highlight a *post mortem* contamination by Fe or Mn in addition to microtomographic analyses to confirm enamel hypomineralisation^37^.

### Microtomographic analyses

The samples including nine discoloured teeth (B335(46), B335(16), S323(26), S323(11), S323(55), S323(65), S323(75), S323(85) and S407(26)) and three control teeth (B335(36), S323(16) and S323(21)), were imaged using high resolution microtomography at the laboratory PLACAMAT (UMS 3626) in Bordeaux, France (Microtomograph X GE™ V/TOME/X S equipment, NY, USA). The scanning parameters were 120 kV, 147 µA for the X-ray tube, exposure time 500 ms with four integrations per projection, 2550 projections/360° and 0.1 mm copper filter placed on the source to reduce beam hardening artefacts. The voxel size was 7 × 7 × 7 μm^3^. The final volume was reconstructed in 16-bit. The microCT images were compiled with the 7.0.1. software Avizo^®^ (Visualization Sciences Group, FEI^®^ Company, OR, USA). In order to take measurements at the same coronal height, images were reconstructed according to the plane of section through two points located at the buccal cervical surface and one point on the lingual cervical surface.

From a selected tooth area, five cubes (≈49 × 49 × 49 µm^3^) were located by means of the *square brush* segmentation tool from the enamel surface to the DEJ (Fig. 4a and SI):

- at the enamel surface (cube n°1);
- at the DEJ (cube n°5).

Then, three equidistant cubes were located:

- cube n°3 at the midway between cubes 1 and 5;
- cube n°2 at the midway between cubes 1 and 3;

- cube n°4 at the midway between cubes 3 and 5.

This process was performed in normal and discoloured enamel. A second round of measures from another selected tooth area was undertaken by the same operator. The mean of the mineral density in each cube was calculated by the function *Measure and Analyse* with Avizo^®^ software. The mineral density difference (mdd) provided in Table 1 corresponds to the *formula*: normal enamel mineral density - discoloured enamel mineral density. To achieve a 3D representation of the MIH defect (Fig. 4a), normal and hypomineralised enamel were segmented by means of the segmentation tool *magic wand* from the Avizo^®^ software. Mineral density profiles (2D; Figs 4b and 5) were analysed using function *Plot Profile* from ImageJ^®^ 1.45 software (NIH, MD, USA). From a slice through affected enamel, the average of grey levels represented by the line from the DEJ to the surface was calculated (Fig. 4b). Along this line for each 7 µm a grey level was recorded. By means of the R statistical software (A language and environment for statistical computing available at https://www.r-project.org/), this line was segmented in 99 equidistant points starting at the DEJ and finishing at the surface (SI). Then, the mean values of these 99 measurements for hypomineralised and normal areas were compared (Fig. 5). Normal enamel measurements from the control group (n = 3) and normal enamel of the discoloured group were determined (n = 8 with the exception of S323(11)). Discoloured enamel measurements were from discoloured enamel from the discoloured group (n = 8 with exception of 321(11)).

### X-ray fluorescence analyses

The samples including nine discoloured teeth (B335(46), B335(16), S323(26), S323(11), S323(55), S323(65), S323(75), S323(85) and S407(26)) were analysed by means of X-Ray fluorescence (Seiko SEA 6000VX, Seiko Corp., Tokyo, Japan) with the following parameters: collimator (0.5 × 0.5 mm), time measurement of 300 s and Rhodium anticathode. Two experimental conditions were used: condition 1 (without filter and tube voltage: 15 kV) to determine relative concentrations of P and Ca, and condition 2 (filter for lead and tube voltage: 50 kV) to determine relative concentrations of Sn, Mn, Cu, Zn, Fe, Pb and Sr. A spot in normal enamel was located at the same coronal height of its homologous spot in stained enamel. This process was repeated a second time. Thus, two measures by area in two distinct zones were performed in stained and normal enamel on the tooth surface. Only chemical elements (in oxide form) that may stain enamel were selected: Mn, Cu, Pb and Fe^70–72^.

### Statistical tests

Non-parametric statistical tests (Wilcoxon test for paired samples) were carried out using Statistica^®^ software Package Version 7.1 (Statsoft Dell, OK, USA) to compare data from normal and discoloured enamel. A Fleiss’s kappa test was performed with R software to test the inter-examiner agreement (n = 19).

## Electronic supplementary material

Supplementary Information

## References

[1] Nanci, A. In Ten Cate’s Oral Histology: *Development, Structure, and Function* Ch. 1, 1–13 (Mosby Elsevier, 2008).

[2] Hillson, S. In *Dental Perspectives on Human Evolution: State of the Art Research in Dental Paleoanthropology* (eds Bailey, S. E. & Hublin, J. J.) xxiii–xxviii (Springer Netherlands, 2007).

[3] Walter BS, DeWitte SN, Redfern RC (2015). Sex differentials in caries frequencies in Medieval London. Arch. Oral Biol..

[4] Goodman AH, Armelagos GJ, Rose JC (1980). Enamel hypoplasias as indicators of stress in three prehistoric populations from Illinois. Hum. Biol..

[5] Clarkson J (1992). A review of the developmental defects of enamel index (DDE Index). Commission on Oral Health, Research & Epidemiology. Report of an FDI Working Group. Int. Dent. J..

[6] Weerheijm KL, Jalevik B, Alaluusua S (2001). Molar-incisor hypomineralisation. Caries Res..

[7] Elfrink ME, Schuller AA, Weerheijm KL, Veerkamp JS (2008). Hypomineralized second primary molars: prevalence data in Dutch 5-year-olds. Caries Res..

[8] Mittal N, Sharma BB (2015). Hypomineralised second primary molars: prevalence, defect characteristics and possible association with Molar Incisor Hypomineralisation in Indian children. Eur. Arch. Paediatr. Dent..

[9] Elfrink ME (2012). Deciduous molar hypomineralization and molar incisor hypomineralization. J. Dent. Res..

[10] Negre-Barber A, Montiel-Company JM, Boronat-Catala M, Catala-Pizarro M, Almerich-Silla JM (2016). Hypomineralized Second Primary Molars as Predictor of Molar Incisor Hypomineralization. Sci. Rep..

[11] Weerheijm KL (2003). Judgement criteria for molar incisor hypomineralisation (MIH) in epidemiologic studies: a summary of the European meeting on MIH held in Athens, 2003. Eur. J. Paediatr. Dent..

[12] Elfrink MEC, Ghanim A, Manton DJ, Weerheijm KL (2015). Standardised studies on Molar Incisor Hypomineralisation (MIH) and Hypomineralised Second Primary Molars (HSPM): a need. Eur. Arch. Paediatr. Dent..

[13] Ghanim A, Morgan M, Marino R, Bailey D, Manton D (2011). Molar-incisor hypomineralisation: prevalence and defect characteristics in Iraqi children. Int. J. Paediatr. Dent..

[14] Jalevik B, Klingberg GA (2002). Dental treatment, dental fear and behaviour management problems in children with severe enamel hypomineralization of their permanent first molars. Int. J. Paediatr. Dent..

[15] Leal, S. C., Oliveira, T. R. & Ribeiro, A. P. Do parents and children perceive molar-incisor hypomineralization as an oral health problem? *Int. J. Paediatr. Dent*., *in press* (2017). 10.1111/ipd.12271 27748991

[16] Dantas-Neta NB (2016). Impact of molar-incisor hypomineralization on oral health-related quality of life in schoolchildren. Braz. Oral Res..

[17] Laisi S, Kiviranta H, Lukinmaa PL, Vartiainen T, Alaluusua S (2008). Molar-incisor-hypomineralisation and dioxins: new findings. Eur. Arch. Paediatr. Dent.

[18] Jedeon K (2013). Enamel defects reflect perinatal exposure to bisphenol A. Am. J. Pathol..

[19] Serna C, Vicente A, Finke C, Ortiz AJ (2016). Drugs related to the etiology of molar incisor hypomineralization: A systematic review. J. Am. Dent. Assoc..

[20] Ghanim A, Manton D, Bailey D, Marino R, Morgan M (2013). Risk factors in the occurrence of molar-incisor hypomineralization amongst a group of Iraqi children. Int. J. Paediatr. Dent..

[21] Whatling R, Fearne JM (2008). Molar incisor hypomineralization: a study of aetiological factors in a group of UK children. Int. J. Paediatr. Dent..

[22] Laisi S (2009). Amoxicillin may cause molar incisor hypomineralization. J. Dent. Res..

[23] Wogelius P (2010). Association between use of asthma drugs and prevalence of demarcated opacities in permanent first molars in 6-to-8-year-old Danish children. Community Dent. Oral Epidemiol..

[24] Vieira AR, Kup E (2016). On the Etiology of Molar-Incisor Hypomineralization. Caries Res..

[25] Kuhnisch J (2014). Genome-wide association study (GWAS) for molar-incisor hypomineralization (MIH). Clin. Oral Investig..

[26] Jeremias F (2013). Genes expressed in dental enamel development are associated with molar-incisor hypomineralization. Arch. Oral Biol..

[27] Silva MJ, Scurrah KJ, Craig JM, Manton DJ, Kilpatrick N (2016). Etiology of molar incisor hypomineralization - A systematic review. Community Dent. Oral Epidemiol..

[28] Arrow P (2009). Risk factors in the occurrence of enamel defects of the first permanent molars among schoolchildren in Western Australia. Community Dent. Oral Epidemiol..

[29] Garot E, Manton D, Rouas P (2016). Peripartum events and molar-incisor hypomineralisation (MIH) amongst young patients in southwest France. Eur. Arch. Paediatr. Dent..

[30] Crombie F, Manton D, Kilpatrick N (2009). Aetiology of molar-incisor hypomineralization: a critical review. Int. J. Paediatr. Dent..

[31] Ogden AR, Pinhasi R, White WJ (2008). Nothing new under the heavens: MIH in the past?. Eur. Arch. Paediatr. Dent..

[32] Curzon ME, Ogden AR, Williams-Ward M, Cleaton-Jones PE (2015). Case report: A medieval case of molar-incisor-hypomineralisation. Br. Dent. J..

[33] Kuhnisch J (2016). Was molar incisor hypomineralisation (MIH) present in archaeological case series?. Clin. Oral Investig..

[34] McKay S, Farah R, Broadbent JM, Tayles N, Halcrow SE (2013). Is it health or the burial environment: differentiating between hypomineralised and post-mortem stained enamel in an archaeological context. PLoS One..

[35] Mansilla J, Solis C, Chavez-Lomeli ME, Gama JE (2003). Analysis of colored teeth from Precolumbian Tlatelolco: postmortem transformation or intravitam processes?. Am. J. Phys. Anthropol..

[36] Stermer EM, Risnes S, Fischer PM (1996). Trace element analysis of blackish staining on the crowns of human archaeological teeth. Eur. J. Oral Sci..

[37] Garot E (2017). Diagnostic guide enabling distinction between taphonomic stains and enamel hypomineralisation in an archaeological context. Arch. Oral Biol..

[38] Turner-Walker, G. In *Advances in Human Palaeopathology* (eds Pinhasi, R. & Mays, S.) p. 11–15 (John Wiley & Sons, Ltd, Chichester, UK., 2007).

[39] Fémolant, J.-M. Beauvais Caserne Taupin “Antenne Universitaire”, Bilan scientifique. *Service Régional de l’Archéologie de Picardie*, p. 48–50 (1992).

[40] Beauval, C. *et al*. *Rapport d’opération de fouilles archéologiques Sains-en-Gohelle* (2012).

[41] Garot E (2016). Mineral density of hypomineralised and sound enamel. Bull. Group. Int. Rech. Sci. Stomatol. Odontol..

[42] Mahoney EK, Rohanizadeh R, Ismail FS, Kilpatrick NM, Swain MV (2004). Mechanical properties and microstructure of hypomineralised enamel of permanent teeth. Biomaterials.

[43] Fearne, J., Anderson, P. & Davis, G. R. 3D X-ray microscopic study of the extent of variations in enamel density in first permanent molars with idiopathic enamel hypomineralisation. *Br. Dent. J*. **196**, 634–638, discussion 625 (2004). 10.1038/sj.bdj.4811282 15153976

[44] Crombie FA (2013). Characterisation of developmentally hypomineralised human enamel. J. Dent..

[45] Farah RA, Swain MV, Drummond BK, Cook R, Atieh M (2010). Mineral density of hypomineralised enamel. J. Dent..

[46] Fagrell TG, Salmon P, Melin L, Noren JG (2013). Onset of molar incisor hypomineralization (MIH). Swed. Dent. J..

[47] Fagrell T (2011). Molar incisor hypomineralization. Morphological and chemical aspects, onset and possible etiological factors. Swed. Dent. J. Suppl..

[48] Ogden AR, Pinhasi R, White WJ (2007). Gross enamel hypoplasia in molars from subadults in a 16th–18th century London graveyard. Am. J. Phys. Anthropol..

[49] Jalevik B, Noren JG, Klingberg G, Barregard L (2001). Etiologic factors influencing the prevalence of demarcated opacities in permanent first molars in a group of Swedish children. Eur. J. Oral Sci..

[50] Beentjes VE, Weerheijm KL, Groen HJ (2002). Factors involved in the aetiology of molar-incisor hypomineralisation (MIH). Eur. J. Paediatr. Dent..

[51] Souza JF (2012). Molar incisor hypomineralisation: possible aetiological factors in children from urban and rural areas. Eur. Arch. Paediatr. Dent..

[52] Aminov RI (2010). A brief history of the antibiotic era: lessons learned and challenges for the future. Front. Microbiol..

[53] Alaluusua S (1996). Developmental dental defects associated with long breast feeding. Eur. J. Oral Sci..

[54] Fagrell TG, Ludvigsson J, Ullbro C, Lundin SA, Koch G (2011). Aetiology of severe demarcated enamel opacities–an evaluation based on prospective medical and social data from 17,000 children. Swed. Dent. J..

[55] Clark, J. F. M. *The Burning Issue: Historical Reflections on Municipal Waste Incineration*. Vol. Short Report (AHRB Research Centre for Environmental History, 2003).

[56] Leppaniemi A, Lukinmaa PL, Alaluusua S (2001). Nonfluoride hypomineralizations in the permanent first molars and their impact on the treatment need. Caries Res..

[57] Dietrich G, Sperling S, Hetzer G (2003). Molar incisor hypomineralisation in a group of children and adolescents living in Dresden (Germany). Eur. J Paediatr. Dent.

[58] Rochester JR, Bolden AL (2015). Bisphenol S and F: A Systematic Review and Comparison of the Hormonal Activity of Bisphenol A Substitutes. Environ. Health Perspect..

[59] Vogel SA (2009). The politics of plastics: the making and unmaking of bisphenol a “safety”. Am. J. Public Health.

[60] Tapias-Ledesma MA (2003). Factors associated with first molar dental enamel defects: a multivariate epidemiological approach. J. Dent. Child..

[61] Lygidakis NA, Dimou G, Marinou D (2008). Molar-incisor-hypomineralisation (MIH). A retrospective clinical study in Greek children. II. Possible medical aetiological factors. Eur. Arch. Paediatr. Dent..

[62] Pitiphat W, Luangchaichaweng S, Pungchanchaikul P, Angwaravong O, Chansamak N (2014). Factors associated with molar incisor hypomineralization in Thai children. Eur. J. Oral Sci..

[63] Ahmadi R, Ramazani N, Nourinasab R (2012). Molar incisor hypomineralization: a study of prevalence and etiology in a group of Iranian children. Iran. J. Pediatr..

[64] Aine L (2000). Enamel defects in primary and permanent teeth of children born prematurely. J. Oral Pathol. Med..

[65] Schaefer, M., Black, S. & Scheuer, L. In *Juvenile Osteology* 67–95 (Academic Press, 2009).

[66] Chevalier, N. *Le cimetière du couvent des Sœurs Grises de Beauvais de la fin du XVe au XVIIIe siècle, Étude des pratiques funéraires* DEA thesis, University of Bordeaux (1993).

[67] Liversidge HM, Marsden PH (2010). Estimating age and the likelihood of having attained 18 years of age using mandibular third molars. Br. Dent. J.

[68] Moorrees CF, Fanning EA, Hunt EEJ (1963). Formation and resorption of three deciduous teeth in children. Am. J. Phys. Anthropol..

[69] Elfrink ME, Veerkamp JS, Aartman IH, Moll HA, Ten Cate JM (2009). Validity of scoring caries and primary molar hypomineralization (DMH) on intraoral photographs. Eur. Arch. Paediatr. Dent..

[70] Tourino LF (2016). Association between Molar Incisor Hypomineralization in Schoolchildren and Both Prenatal and Postnatal Factors: A Population-Based Study. PLoS One..

[71] Brook AH, Smith RN, Lath DJ (2007). The clinical measurement of tooth colour and stain. Int. Dent. J.

[72] Watts A, Addy M (2001). Tooth discolouration and staining: a review of the literature. Br. Dent. J..

