# Supplementary material for: Analytical evidence of enamel hypomineralisation on permanent and primary molars amongst past populations
Source: Sci Rep. 2017 May 10;7:1712. doi: 10.1038/s41598-017-01745-w (PMC5431767; doi:10.1038/s41598-017-01745-w)
Supplement: Supplementary file 1 — Supplementary Information [file 41598_2017_1745_MOESM1_ESM.pdf]

Analytical evidence of enamel hypomineralisation on permanent and primary molars amongst past populations

Elsa Garot*[a,b], Christine Couture-Veschambre[b], David Manton[c], Cédric Beauval[d], Patrick Rouas[a,b]

[a]Univ. de Bordeaux, UFR des Sciences Odontologiques, Bordeaux, France
[b]Univ. de Bordeaux, PACEA, UMR 5199, Pessac, France
[c]Melbourne Dental School, University of Melbourne, Victoria, Australia
[d]Archéosphère, 2 Rue des Noyers, 11500 Quirbajou, France

*Corresponding author

Correspondence to elsa.garot@u-bordeaux.fr,

Supplementary Table 1. Details of MIH specialists' responses for each specimen (a) and kappa Fleiss' test performed between responses (B335, S323 and S407) to test agreement degree on clinical diagnosis of MIH.

a)	B335	S323	S407
1	Yes	Yes	Yes
2	Unsure	Unsure	Unsure
3	Unsure	Unsure	Yes
4	Yes	Yes	Yes
5	Yes	No	Yes
6	Yes	No	Yes
7	Yes	Yes	Yes
8	Yes	Yes	Yes
9	No	Unsure	Unsure
10	Unsure	Yes	Yes
11	Yes	Yes	Yes
12	Yes	No	Yes
13	Yes	Yes	Unsure
14	Unsure	No	No
15	Yes	Unsure	Yes
16	Yes	Unsure	Yes
17	Unsure	No	Yes
18	Unsure	Unsure	No
19	Unsure	Yes	Yes

b) Cases		Yes	No	Unsure	Total	Fleiss's κ
B335	(n ; %)	11; 58	1; 5	7; 37	19; 100	<0
S323	(n ; %)	8; 42	5; 26	6; 32	19; 100	<0
S407	(n ; %)	14; 74	2; 11	3; 16	19; 100	<0
						0.0052

Supplementary Table 2. 3D data and mineral densities determined by microcomputed tomography analyses. GL, Grey level ; **1**, enamel surface; **2,** midway between 1 and 3; **3**, midway between 1 and 5; **4** midway between 3 and 5; **5**, enamel dentin junction; m, mean; SD, standard deviation; mdd, mineral density difference.

Tooth	Area	Analyse 1 (GL)					Analyse 2 (GL)				
		**1**	**2**	**3**	**4**	**5**	**1**	**2**	**3**	**4**	**5**
B335(46)	Discoloured	51143.27	46627.82	48078.45	47143.20	45922.02	48946.15	48137.52	47196.66	47089.55	44734.33
B335(16)	Discoloured	50349.05	46191.46	45172.16	44127.30	44138.89	49943.82	46755.06	45488.54	45205.63	43427.42
S323(55)	Discoloured	52301.95	45768.67	45644.54	48178.66	47140.19	53878.00	45673.71	44778.18	46429.14	44958.87
S323(65)	Discoloured	53641.84	51449.97	46978.83	44769.32	46038.72	52921.17	50063.78	43944.25	42697.41	44868.25
S323(75)	Discoloured	54060.75	46491.25	44529.52	45572.43	45495.46	53074.51	47340.52	41624.81	44032.04	47811.14
S323(85)	Discoloured	52388.45	45564.59	42210.57	43609.63	45531.71	53753.18	50000.20	46371.78	46057.32	46204.30
S323(11)	Discoloured	49422.32	50499.43	51257.05	51957.19	51568.27	57626.29	52170.31	51733.62	51671.83	50858.36
S323(26)	Discoloured	53814.66	47308.77	44984.29	43182.63	43718.72	53212.95	44836.43	42338.43	43380.83	43658.92
S407(26)	Discoloured	47619.87	43435.07	46151.47	45960.82	45248.82	48125.11	43589.09	46336.70	45874.63	43745.10
**m**	**Discoloured**	**51638.02**	**47037.45**	**46111.88**	**46055.69**	**46089.20**	**52386.80**	**47618.51**	**45534.77**	**45826.49**	**45585.19**
SD	*Discoloured*	*2196.24*	*2486.65*	*2529.58*	*2747.07*	*2289.86*	*2935.82*	*2766.34*	*2982.03*	*2635.34*	*2411.62*
B335(46)	Normal	55224.77	51081.28	49531.41	47883.66	45564.79	55983.70	52668.33	51519.46	49932.82	47129.98
B335(16)	Normal	54420.50	50139.23	48509.47	46727.19	46438.95	50294.45	48213.03	46851.20	46099.88	45594.21
S323(55)	Normal	54401.00	51567.79	49572.19	48696.99	48715.41	54449.14	51377.32	48938.70	48140.23	47549.47
S323(65)	Normal	54998.55	53102.39	51691.18	50562.35	49703.87	54461.06	50326.05	48163.60	47870.34	46747.41
S323(75)	Normal	54477.51	50291.41	48157.63	48040.41	47018.32	55403.86	50801.39	47635.05	46584.65	45985.59
S323(85)	Normal	54247.43	50121.52	48318.83	48591.45	48125.59	55476.08	50475.83	47941.99	47856.75	47575.47
S323(11)	Normal	50481.35	51328.84	51013.67	50577.61	49940.02	50747.20	51609.20	51206.11	50757.74	50074.84
S323(26)	Normal	53874.34	49637.61	47623.47	45736.30	44731.47	54864.59	51009.50	49631.85	48992.71	46970.84
S407(26)	Normal	53000.55	51322.56	50579.72	49338.69	47569.98	52519.87	50544.39	49721.64	48964.37	46876.59
B335(26)	Normal	55307.95	50779.75	48752.82	47588.50	45845.44	53644.93	50154.45	49179.53	48241.74	46026.47
B335(26)	Normal	54577.41	50581.55	49199.96	48364.82	45461.69	53816.46	49991.64	48366.35	47658.84	45534.35
S323(16)	Normal	54739.91	51240.03	49727.11	48258.73	45842.09	52812.76	49755.30	48030.42	47080.08	46452.01
S323(16)	Normal	53710.98	50517.12	49179.14	47960.49	45975.82	54076.75	51078.34	49214.39	48399.53	46574.69
S323(21)	Normal	56283.36	53635.29	51755.77	50826.96	49770.09	52889.20	51829.55	50979.10	50442.19	49787.56
S323(21)	Normal	56220.79	53853.04	52350.04	51481.92	49537.88	52169.61	51122.83	50803.54	49904.56	50153.93
**m**	**Normal**	**54397.76**	**51279.96**	**49730.83**	**48709.07**	**47349.43**	**53573.98**	**50730.48**	**49212.20**	**48461.76**	**47268.89**
SD	*Normal*	*1387.89*	*1288.84*	*1443.68*	*1593.33*	*1820.17*	*1673.02*	*1033.06*	*1423.07*	*1375.84*	*1544.58*
mdd		2759.74	4242.51	3618.95	2653.38	1260.23	1187.18	3111.96	3677.42	2635.28	1683.71
mdd (%)		5.1	8.3	7.3	5.4	2.7	2.2	6.1	7.5	5.4	3.6
mdd mean (%)	Analyses 1&2	**3.6**	**7.2**	**7.4**	**5.4**	**3.1**					

Supplementary Figure 1. Selected coronal and sagittal slides provided by microcomputed tomography for 2D analyses of mineral densities line profiles by microcomputed tomography analyses. a, B335(46); b, B335(36); c, B335(16); d, S323(55); e, S323(85); f, S323(65); g, S323(75); h, S323(26); i, S323(16); j, S323(21); k, S407(26); ★ affected area.

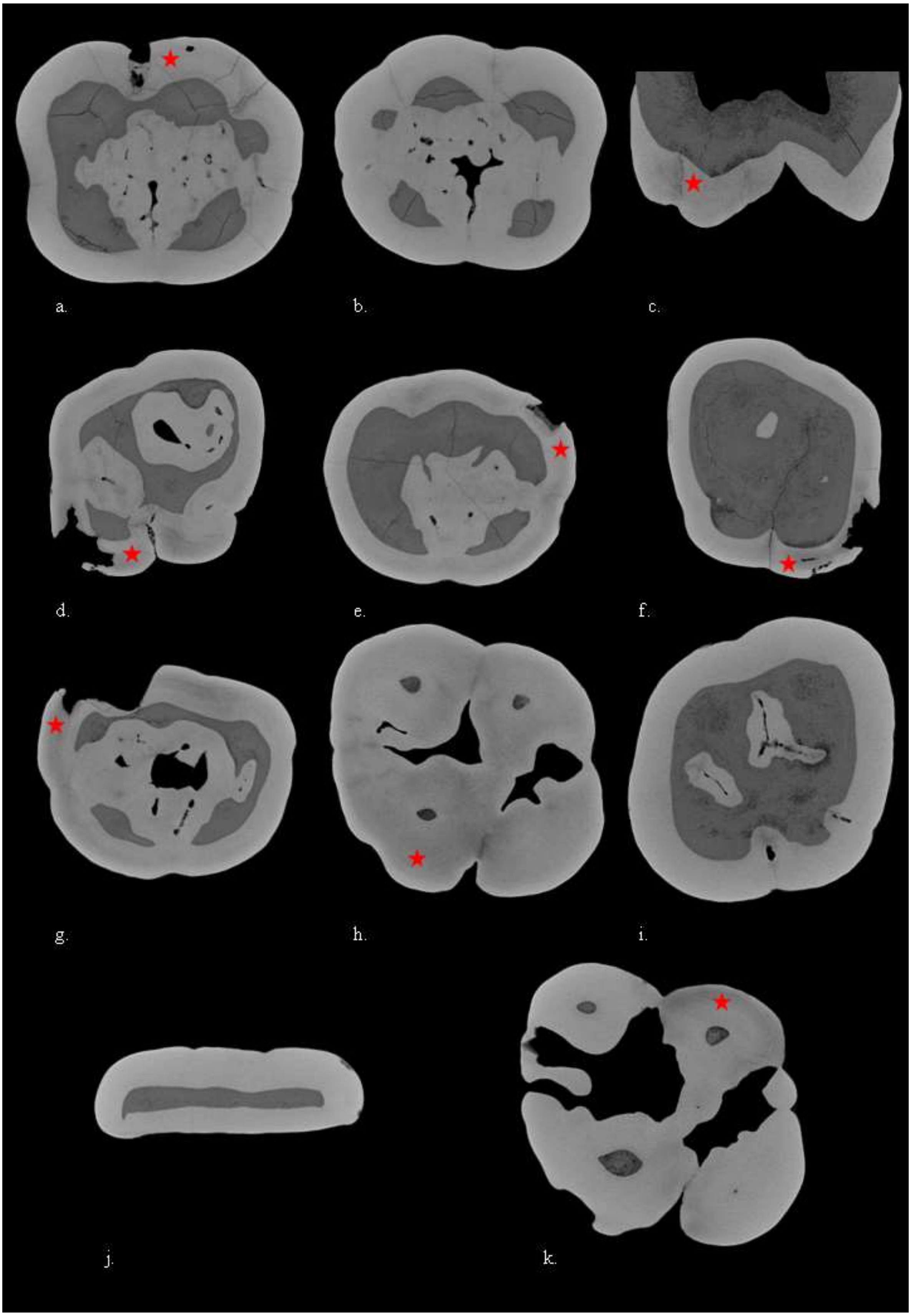

Supplementary Table 3.2D data and line profiles of mineral densities from DEJ to surface (99 equidistant measurements) along the lines AB and CD from DEJ to surface (table 4b) provided by microcomputed tomography analyses. Normal enamel measurements come from control group (n = 3) and normal enamel of discoloured group (n = 8 with the exception of S323(11)). Discoloured enamel measurements come from discoloured enamel from discoloured group (n = 8 with exception of 321(11)). DEJ, Dento-Enamel Junction; GL, Grey level ; m, mean; SD, standard deviation; mdd, mineral density difference.

m normal (GL)	SD (GL)	m discoloured (GL)	SD (GL)	mdd (GL)	mdd (%)
46305.59	2949.20	44793.58	3728.48	1512.01	3.27
46300.70	3678.45	45414.27	3090.19	886.43	1.91
46023.30	3174.19	45472.40	2901.50	550.90	1.20
47098.10	2331.05	45727.00	2771.92	1371.10	2.91
46984.93	3838.21	46603.25	2850.31	381.68	0.81
47977.89	3145.46	46444.81	2465.67	1533.08	3.20
48285.67	2943.33	47504.89	2362.53	780.78	1.62
48713.22	3049.03	46951.91	4124.83	1761.31	3.62
47948.15	3924.97	44292.75	4370.03	3655.40	7.62
46750.56	3118.77	46428.32	5420.01	322.24	0.69
46619.36	3156.68	46322.40	3238.06	296.96	0.64
47155.99	2353.00	44764.80	2107.35	2391.19	5.07
47360.43	2885.50	44568.08	3592.90	2792.35	5.90
46688.63	2523.18	45930.27	4304.09	758.36	1.62
47021.86	3298.54	46339.14	2417.11	682.72	1.45
47424.73	2882.56	45277.43	2500.11	2147.30	4.53
50107.50	3326.52	45897.65	4435.63	4209.85	8.40
48207.74	2543.64	46967.90	3810.53	1239.84	2.57
46406.75	2573.35	47545.07	3191.60	-1138.32	-2.45
47840.10	2591.68	46071.03	2249.50	1769.07	3.70
47855.99	2960.26	47273.94	4271.48	582.05	1.22
47062.25	3955.59	47273.28	3743.90	-211.03	-0.45
47478.94	2766.23	46790.65	1741.28	688.29	1.45
47569.70	1968.96	44450.87	2532.60	3118.83	6.56
47842.86	3420.09	45656.04	2827.31	2186.82	4.57
47716.06	2052.54	45342.86	3464.51	2373.20	4.97
48109.47	2394.21	47267.59	1927.40	841.88	1.75
47178.75	3560.57	46561.33	4108.10	617.42	1.31
49472.29	2830.70	45184.63	3054.36	4287.66	8.67
48726.81	2920.78	44866.53	3228.93	3860.28	7.92
48999.19	2463.77	44527.21	5830.03	4471.98	9.13
47624.86	3031.62	46459.81	3133.36	1165.05	2.45
46649.97	2441.73	44374.37	3313.17	2275.60	4.88
48912.65	2385.31	45069.74	2333.31	3842.91	7.86
48409.41	3685.29	46663.51	3797.24	1745.90	3.61
46773.81	3982.12	43191.82	3381.26	3581.99	7.66
49039.11	2611.77	43036.96	4002.99	6002.15	12.24
48966.95	3296.41	43001.40	2626.29	5965.55	12.18

48683.84	2278.65	45936.03	3457.02	2747.81	5.64
48272.22	1861.41	46237.60	2778.10	2034.62	4.21
48590.60	3052.66	47695.76	3583.73	894.84	1.84
48379.47	2652.36	45288.49	5164.17	3090.98	6.39
49689.30	3505.14	46676.13	3103.02	3013.17	6.06
48910.98	2738.76	44167.07	3356.34	4743.91	9.70
48561.14	3320.12	45580.08	4064.15	2981.06	6.14
48045.73	2841.23	44635.93	2968.89	3409.80	7.10
47150.74	3914.17	46299.90	3030.25	850.84	1.80
48879.71	2726.25	44711.52	2819.89	4168.19	8.53
49272.73	2183.94	44643.62	3055.88	4629.11	9.39
48384.61	3334.05	44018.51	3766.92	4366.10	9.02
49426.87	2341.06	44770.20	3436.86	4656.67	9.42
49344.20	3465.74	43686.17	2473.80	5658.03	11.47
50017.35	2466.36	45057.49	1802.71	4959.86	9.92
49887.67	1543.04	44896.68	1298.82	4990.99	10.00
49358.23	1944.08	42587.12	3425.97	6771.11	13.72
47979.80	2548.72	44494.46	2160.93	3485.34	7.26
48708.17	3106.48	44875.43	3666.32	3832.74	7.87
50334.47	2043.82	44690.07	2231.16	5644.40	11.21
50244.19	2919.26	45193.60	3882.82	5050.59	10.05
49969.19	4105.61	46595.61	1236.49	3373.58	6.75
50410.27	2311.91	45973.71	3122.46	4436.56	8.80
50873.79	2345.98	45966.46	2256.51	4907.33	9.65
49879.13	2244.43	46195.57	3191.56	3683.56	7.38
49189.72	2503.64	46002.50	2207.97	3187.22	6.48
49704.71	3235.53	43189.09	2635.66	6515.62	13.11
48189.28	2907.80	46093.59	2015.52	2095.69	4.35
50198.80	3226.61	45171.58	2408.65	5027.22	10.01
50405.97	3210.33	46233.17	1739.42	4172.80	8.28
49734.63	3030.15	45504.12	2229.06	4230.51	8.51
50307.63	2601.35	43774.37	3335.70	6533.26	12.99
50702.88	2354.31	45063.82	1573.88	5639.06	11.12
51845.64	1718.68	46190.65	3253.01	5654.99	10.91
49761.31	4177.14	46639.51	3324.54	3121.80	6.27
52208.63	3237.08	45397.08	3987.63	6811.55	13.05
51580.43	2633.11	46675.40	2278.47	4905.03	9.51
50493.42	1259.54	46307.90	2771.29	4185.52	8.29
51709.14	3335.14	45671.55	4133.20	6037.59	11.68
50522.06	3228.79	47467.08	3410.43	3054.98	6.05
50568.04	2923.15	45753.39	2108.18	4814.65	9.52
51320.16	2027.32	47413.31	3283.21	3906.85	7.61
51616.88	2219.81	47220.20	2806.07	4396.68	8.52
53996.55	1997.42	48500.53	2465.95	5496.02	10.18

52805.04	2035.66	47570.71	4613.27	5234.33	9.91
52941.29	2523.70	47964.95	3275.31	4976.34	9.40
52803.45	4143.12	47564.50	2735.18	5238.95	9.92
52449.42	2821.94	48531.15	3313.10	3918.27	7.47
52754.22	3696.12	49864.67	3890.35	2889.55	5.48
52074.15	2560.10	48969.40	1964.72	3104.75	5.96
54011.19	2260.04	49673.25	2893.13	4337.94	8.03
52814.98	2216.16	49845.46	4901.38	2969.52	5.62
51690.69	2128.35	48713.33	2512.01	2977.36	5.76
52980.35	2736.04	48815.04	2759.49	4165.31	7.86
54407.13	3344.92	49179.68	3008.94	5227.45	9.61
54021.19	3431.26	50677.91	2935.49	3343.28	6.19
53754.72	2831.92	52489.12	1852.62	1265.60	2.35
54518.67	3377.55	50245.78	1183.53	4272.89	7.84
55136.10	2114.06	51499.27	2897.40	3636.83	6.60
55355.35	2651.18	53297.13	2786.68	2058.22	3.72
57038.04	2573.35	50816.02	4906.81	6222.02	10.91

Supplementary data. Archaeological information on series data

The convent "Les soeurs Grises"

In 1992, an exhaustive excavation of the site conducted by J.M. Fémolant helped uncover more than 350 burials and 50 ossuaries and collective burials. The convent was occupied by the religious community between 1480 and the late 18th century. From 1630, their main activity was child education entrusted by Beauvais families. In 1502, the community got to the convent the right to bury. The cemetery received his first relics the 20 August 1505.

Sains-en-Gohelle

The cemetery would have been utilised for nearly a millennium from the late 7th century to the early 17th century. The buried individuals correspond to a parish population, even if funerary recruitment may change over time. The size of the cemetery (more than 1500 people on 1100 m^2 searched by two scientific teams) is very important in terms of archival data.

References

Chevalier, N. *Le cimetière du couvent des Sœurs Grises de Beauvais de la fin du XVe au XVIIIe siècle, Étude des pratiques funéraires* DEA thesis, University of Bordeaux, (1993).

Beauval, C. *et al. Rapport d'opération de fouilles archéologiques Sains-en-Gohelle*. (2012).
